# Novel 2-(ω-phosphonooxy-2-oxaalkyl)acrylate monomers for self-etching self-priming one part adhesive

**DOI:** 10.3762/bjoc.6.95

**Published:** 2010-09-07

**Authors:** Joachim E Klee, Uwe Lehmann

**Affiliations:** 1 Dentsply De Trey, De-Trey-Str. 1, 78467 Konstanz, Germany

**Keywords:** adhesion to enamel and dentin, hydrolysis stable 2-(ω-phosphonooxy-2-oxaalkyl)acrylates, phosphorylation using POCl_3_, polymerization enthalpy, shear bond strength

## Abstract

Novel hydrolysis stable 2-(ω-phosphonooxy-2-oxaalkyl)acrylate monomers **3** with phosphoric acid moieties were synthesized by a three step synthesis via Baylis–Hillman reaction of ethyl acrylate and formaldehyde, and subsequent etherification of the obtained product with diols and phosphorylation using POCl_3_. The polymerization enthalpy of 2-(ω-phosphonooxy-2-oxaalkyl)acrylates **3** as measured by DSC ranges from −29 to −53 kJ**·**mol^−1^. The shear bond strength of adhesive compositions **4,** comprising of polymerizable acids **3,** ranges from 5.8 to 19.3 MPa on enamel and from 8.7 to 16.9 MPa on dentin.

## Introduction

Dental adhesives have been employed for fixation of direct and indirect restorations since the 80s of the last century. The first of these adhesives were composed of three-part systems, consisting of an etch gel, a primer and a bonding. Each of these adhesive parts was applied step-by-step in a relatively time consuming procedure that is prone to failure during the procedure. Therefore, a reduction of the complexity of the adhesives during application was desirable. Furthermore, the adhesion procedure should be more safe, easy and robust. In order to fulfill these demands some generations of adhesives were developed which combined the etch and prime function or the prime and bond function together in one part.

The first self-etching, self-priming dental adhesives were composed of two-part systems for stability reasons, i.e., low hydrolysis stability of conventional polymerizable acidic ester monomers and adhesive monomers in water or water/solvent mixtures. Newer one-part systems are based on conventional polymerizable acidic ester monomers and adhesive monomers, and these must be stored in a refrigerator to guarantee stability.

Today the demand is to have a one-part self-etching adhesive, which can be stored under ambient conditions and which combines all three steps of the adhesive procedure in one. Besides polymerizable monomers and acidic monomers, water is required in such a system for the acidic etch function. Therefore, such adhesives require novel hydrolysis stable monomers and novel hydrolysis stable acidic monomers.

Recently, some interesting approaches to acidic monomers were developed based on derivatives of α-hydroxymethylacrylate esters [1], with one [2–4] or two phosphonic acid moieties [5], or with phosphoric [4,6] or sulfonic acid groups [7] as well as with carboxylic acid groups [7]. These acidic monomers are constructed in such a way that no hydrolysis sensitive moiety is present between the polymerizable moiety and the acidic group. The latter etches enamel and dentin surface, and is an anchor group, especially on dentin, due to the formation of calcium phosphate linkages.

The aim of the present investigation is the synthesis of novel polymerizable phosphoric acid ester monomers and their comparison concerning their adhesive performance in dental formulations. Furthermore, the aim is to clarify whether, under strong acidic conditions, hydrolysis of phosphoric acid ester moieties takes place in a similar manner to carboxylic acid esters, and to establish whether acidic molecules with 2-(ω-phosphonooxy-2-oxaalkyl)acrylate moieties exhibit the same adhesive performance as their ester analogues.

## Results and Discussion

**Scheme 1:**
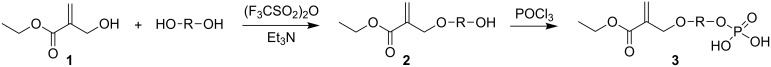
Synthesis of novel ethyl 2-(ω-phosphonooxy-2-oxaalkyl)acrylates **3**.

A series of novel 2-(ω-phosphonooxy-2-oxaalkyl)acrylates **3,** comprising of phosphoric acid ester moieties, were synthesized via a three step synthesis via Baylis–Hillman reaction of ethyl acrylate and formaldehyde, and subsequent etherification of the obtained product with diols and phosphorylation using POCl_3_ (Scheme 1, Table 1).

**Table 1:** Yields, viscosities and refraction indexes of molecules **2a–2i** and **3a–3i**.

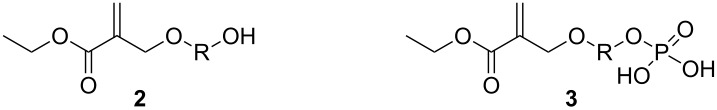							

	R		*M*_n_ g/mol	Yield **2** g (%)	Yield **3** g (%)	η Pa*s	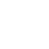

a	(CH_2_)_2_	C_8_H_15_O_7_P	254.18	10.12 (50.0)	14.2 (55.0)	40.13 ± 0.62	1.4760 ± 0.002
b	(CH_2_)_3_	C_9_H_17_O_7_P	268.20	37.28 (21.4)	31.0 (58.8)	4.01 ± 0.10	1.4662 ± 0.001
c	(CH_2_)_6_	C_12_H_23_O_7_P	310.28	16.01 (36.2)	8.8 (44.5)	2.04 ± 0.05	1.4652 ± 0.0003
d	(CH_2_)_10_	C_16_H_31_O_7_P	366.39	40.49 (40.9)	11.5 (20.9)	-	-
e	(CH_2_)_10_^a^	C_14_H_27_O_7_P	338.34	-	8.6 (60.0)	-	-
f	(CH_2_)_12_	C_18_H_35_O_7_P	394.45	7.07 (8.8)	7.8 (91.0)	-	-
g	CH_2_CH_2_OCH_2_CH_2_	C_10_H_19_O_8_P	298.23	19.45 (46.0)	17.0 (65.0)	6.26 ± 0.16	1.4691 ± 0.002
h	(CH_2_CH_2_O)_2_CH_2_CH_2_	C_12_H_23_O_9_P	342.28	28.34 (46.9)	31.1 (88.6)	4.16 ± 0.05	1.4692 ± 0.0002
i	(CH_2_CH_2_O)_3_CH_2_CH_2_	C_14_H_27_O_10_P	386.34	51.91 (73.5)	32.9 (50.2)	2.35 ± 0.01	1.4702 ± 0.0003

^a^**3e** with free carboxylic acid moiety.

The double bonds in **3** are evident in the IR spectrum at 1637 (**3a**) and 1639 cm^−1^ (**3e, 3h, 3i**) and in the ^1^H NMR spectrum at 5.87/6.27 ppm (**3a**). In the ^13^C NMR spectra signals of the sp^2^ hybridized C-atoms of the double bonds appear at 127.11/136.281 ppm (**3a**). Signals of the P–OH moiety were found in the ^1^H NMR spectrum at 10.71 ppm (**3a**) and in the ^31^P NMR spectra at 0.16 ppm (**3h**) and −0.11/0.07 ppm (**3i**).

### DSC Investigation of 3

The polymerization behavior of 2-(ω-phosphonooxy-2-oxaalkyl)acrylates **3** was investigated by photo-polymerization using a DSC 7/DPA 7 unit. The polymerization enthalpy of 2-(ω-phosphonooxy-2-oxaalkyl)acrylates **3** (Table 2) ranged from −29 to −53 kJ**·**mol^−1^ per double bond, which is rather comparable to the polymerization enthalpy of methacrylic esters (−52.8 to −59.9 kJ**·**mol^−1^) [8–10] and lower compared to the polymerization enthalpy of acrylic esters (−77.5 to −80.5 kJ**·**mol^−1^) [9–10]. This may be due to the substitution of the double bond in **3,** which is comparable with a methacrylic moiety rather than with an acrylic group.

**Table 2:** Polymerization enthalpy Δ_R_*H* of **3a–3i**.

**3**	R	Δ_R_*H* kJ/mol

a	(CH_2_)_2_	−30.7
b	(CH_2_)_3_	−45.7
c	(CH_2_)_6_	−44.8
d	(CH_2_)_10_	−45.1
e	(CH_2_)_10_^a^	−53.2
f	(CH_2_)_12_	−52.0
g	CH_2_CH_2_OCH_2_CH_2_	−29.3
h	(CH_2_CH_2_O)_2_CH_2_CH_2_	−41.8
i	(CH_2_CH_2_O)_3_CH_2_CH_2_	−50.8

^a^**3e** with free carboxylic acid moiety.

Obviously, molecules **3** with relatively short alkyl or oxyalkyl spacers exhibit the lowest polymerization enthalpy of −30.7 (**3a**) and −29.3 kJ**·**mol^−1^ (**3g**). The longer the spacers are, the higher is the polymerization enthalpy, up to −50.8 (**3i**) and −52.0 kJ**·**mol^−1^ (**3f**), which is only slightly lower than those of methacrylates. Probably, the steric proximity of the phosphoric acid moiety has an influence on the polymerization behavior due to the competitive reactions of protonation and radical formation of the amine co-initiator.

Assuming that there was complete polymerization at 80 °C, the polymerization enthalpy of **3b** was measured under these conditions. It was found that the polymerization enthalpy increases from −45.7 at 37 °C to −52.3 kJ/mol at 80 °C, which corresponds to a degree of double bond conversion of 87.3% at 37 °C.

### Adhesion of phosphoric acids 3

**Figure 1:**
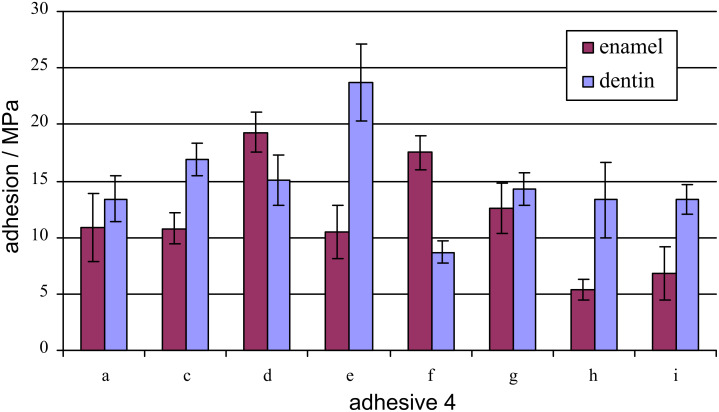
Adhesion of an adhesive composition **4,** comprising of polymerizable acids **3,** on enamel and dentin.

The shear bond strength of adhesive compositions **4** [11], comprising of polymerizable acids **3,** ranges from 5.8 to 19.3 MPa on enamel and from 8.7 to 16.9 MPa on dentin (Table 3, Figure 1). With increasing length of the aliphatic spacers of **3a**–**3f** between the phosphoric acid ester and the ether linkage, adhesion both on enamel and dentin reach a maximum at **3e** (*n* = 10 CH_2_ moieties). Obviously, a balance of the hydrophobic and hydrophilic nature is achieved with **3e,** which is essential for adhesion on the very different substrates such as enamel and dentin.

Whilst the inclusion of larger oxymethylene moieties in **3** has no influence on the adhesion on dentin (**3a**, **3g**–**3h**), it has a pronounced effect on the adhesion on enamel which drops significantly with larger numbers of oxymethylene moieties.

**Table 3:** Adhesion of an adhesive composition **4,** comprising of polymerizable acids **3,** on enamel and dentin.

	Adhesion on	
**4**	enamel/MPa	dentin/MPa

a	10.9 ± 3.0	13.4 ± 2.0
c	10.8 ± 1.4	16.9 ± 1.4
d	19.3 ± 1.8	15.1 ± 2.2
e	10.5 ± 2.4	23.7 ± 3.4
f	17.5 ± 1.5	8.7 ± 1.0
g	12.6 ± 2.2	14.3 ± 1.4
h	5.4 ± 0.9	13.3 ± 3.4
i	6.8 ± 2.4	13.4 ± 1.3

## Conclusion

Novel hydrolysis stable 2-(ω-phosphonooxy-2-oxaalkyl)acrylate monomers **3** with phosphoric acid moieties and alkyl as well as oxyalkyl spacers were synthesized in three steps via Baylis–Hillman reaction of ethyl acrylate and formaldehyde, and subsequent etherification of the obtained product with diols and phosphorylation using POCl_3_. The polymerization enthalpy of 2-(ω-phosphonooxy-2-oxaalkyl)acrylates **3** measured by DSC ranges from −29 to −53 kJ**·**mol^−1^. The shear bond strength of adhesive compositions **4,** comprising of polymerizable acids **3,** ranges from 5.8 to 19.3 MPa on enamel and from 8.7 to 16.9 MPa on dentin.

## Experimental

The following commercial products were purchased from Sigma-Aldrich: formalin, ethyl acrylate, diazabicyclo[2.2.2]octane, dioxane, trifluoromethane sulphonic anhydride, dichloromethane, conc. HCl, triethylamine, 1,2-ethanediol, 1,6-hexanediol, 1,8-octanediol 1,10-decanediol, 1,12-dodecanediol, diethylene glycol, triethylene glycol, tetraethylene glycol, phosphorous oxychloride, diethyl ether and 2,6-di-*tert*-butyl-4-methylphenol Bis(2,4,6-trimethylbenzoyl)phenyl phosphine oxide was purchased from BASF Interorgana. *N*,*N*´-bisacrylamido-*N*,*N*´-diethyl-1,3-propane, and 3(4)8(9)-bis(acrylamidomethyl)tricyclo[5.2.1.0^2,6^]decane were obtained from DENTSPLY. Spectrum TPH (DENTSPLY) is a dental composite comprising of methacrylate based resins and a glass filler.

### 

#### Ethyl 2-hydroxymethylacrylate (1)

**1** was prepared according to [1].

#### Ethyl 2-(4-hydroxy-2-oxabutyl)acrylate (2a)

To a solution of 32.52 g trifluoromethane sulphonic anhydride in 100 mL dichloromethane, a solution of 15 g (0.115 mol) **1** and 11.66 g (0.115 mol) triethylamine in 200 mL dichloromethane was added slowly at such a rate that the temperature of the reaction mixture remained below 5 °C. The solution was then added dropwise at room temperature to 210 g (3.387 mol) 1,2-ethanediol. After stirring for 12 h at room temperature, the solution was washed successively with 1 × 200 mL water, 2 × 250 mL of an aqueous sodium carbonate solution (25 wt %) and 1 × 200 mL water. The organic layer was dried over magnesium sulfate and filtered. After the evaporation of the solvent, the oily crude product was stabilized with 15 mg 2,6-di-*tert*-butyl-4-methylphenol and purified by vacuum distillation (63 °C/0.032 mbar) to give 10.12 g (yield: 50%) of **2a** as a clear, colorless oil.

IR (film, cm^−1^) 3436 (OH), 2979, 2931, 2871 (CH_3_/CH_2_), 1710 (CO), 1638 (C=C), 1453, 1373, 1304 (CH_3_/CH_2_), 1270, 1173, 1109, 1052, 953. ^1^H NMR (250 MHz, CDCl_3_, ppm) 1.27 (t, 3H, CH_3_), 2.52 (broad s, 1H, OH), 3.54–3.63 (m, 2H, OC*H_2_*CH_2_O), 3.67–3.78 (m, 2H, OC*H*_2_CH_2_O), 4.15–4.24 (m, 4H, CH_2_ (1) and OC*H*_2_CH_3_), 5.84 (s, 1H, CH*=C*), 6.28 (s, 1H, CH=C). ^13^C NMR (63 MHz, CDCl_3_, ppm) 14.06 (CH_3_), 60.74 (O*C*H_2_CH_3_), 61.56 (*C*H_2_OH), 69.31 (*C*H_2_CH_2_OH), 71.80 (=C*C*H_2_O), 126.21 (*C*H_2_=C), 137.03 (*C*=CH_2_), 165.82 (CO).

#### Ethyl 2-(4-phosphonooxy-2-oxabutyl)acrylate (3a)

To a stirred solution of 15.46 g (0.1008 mol) phosphorus oxychloride in 280 mL diethyl ether, a solution of 17.56 g (0.1008 mol) **2a** and 10.2 g (0.1008 mol) triethylamine in 250 mL diethyl ether was added dropwise at such a rate that the temperature of the reaction mixture remained below 5 °C. After stirring for 14 h at room temperature, the mixture was filtered and then added slowly at 0 °C to 200 mL of water. The emulsion was stirred for 40 min. The layers were separated and the aqueous layer was washed with 2 × 100 mL diethyl ether. The aqueous layer was reduced in volume to 100 mL and extracted with 4 × 100 mL dichloromethane. The combined organic extracts were dried over magnesium sulfate, filtered and evaporated to yield 19 g of a yellow oil. The oil was dissolved in 400 mL of water and washed with 3 × 200 mL of diethyl ether. Concentration of the aqueous phase at reduced pressure and drying under vacuum (10^−3^mbar) afforded 14.18 g (yield: 55%) of a clear colorless oil, which was stabilized with 6.11 mg 2,6-di-*tert*-butyl-4-methylphenol.

IR (film, cm^−1^) 3500–2500 broad absorption (OH), 2912 (CH_3_/CH_2_), 1709 (CO), 1637 (C=C), 1456, 1374 (CH_3_/CH_2_), 1261, 1178, 1105, 1014, 949. ^1^H NMR (250 MHz, CDCl_3_, ppm) 1.25 (t, 3H, CH_3_), 3.70 (broad s, 2H, CH_2_), 4.30–4.13 (m, 6H, CH_2_), 5.87 (s, 1H, CH=C), 6.27 (s, 1H, CH=C), 10.71 (broad s, 2 H, PO_3_H_2_). ^13^C NMR (63 MHz, CDCl_3_, ppm) 13.94 (CH_3_), 60.94 (O*C*H_2_CH_3_), 66.01 (OCH_2_*C*H_2_), 69.25 (O*C*H_2_CH_2_), 69.40 (=C*C*H_2_O), 127.11 (*C*H_2_=C), 136.28 (*C*=CH_2_), 166.00 (CO). ^31^P NMR (CD_3_OD; ppm): −0.21/0.01 (d, PO_3_H_2_).

**2b**–**2i** and **3b**–**3i** were synthesized as described for **2a** and **3a**.

#### Ethyl 2-(5-hydroxy-2-oxapentyl)acrylate (2b)

bp 100–120 °C/0.093 mbar. IR (film, cm^−1^): 3500–2500 broad absorption (OH), 2877 (CH_3_/CH_2_), 1705 (CO), 1637 (C=C), 1469, 1381, 1177, 1101, 1002, 953, 819, 746. ^1^H NMR (250 MHz, *d*_6_-DMSO, ppm): δ = 1.18 (t, 3H, CH_3_), 1,72–1,87 (m, 2H, CH_2_), 3.43–3,51 (m, 2H, CH_2_), 3.81–3,94 (m, 2H, CH_2_), 4.00–4.19 (m, 4H, CH_2_), 5.79 (s, 1H, CH=C), 6.12 (s, 1H, CH=C). ^13^C NMR (63 MHz, *d*_6_-DMSO, ppm): δ = 14.35 (CH_3_), 30.58, 30.65 (CH_2_*C*H_2_CH_2_), 60.79 (O*C*H_2_CH_3_), 63.15, 63.18 (O*C*H_2_CH_2_), 66.88 (O*C*H_2_CH_2_), 68.76 (=C*C*H_2_O), 125.77 (*C*H_2_=C), 137.84 (*C*=CH_2_), 165.61 (CO).

#### Ethyl 2-(8-hydroxy-2-oxaoctyl)acrylate (2c)

The substance was dried in vacuum without distillation. IR (film, cm^−1^): 3383 (OH), 2935/2866 (CH_3_/CH_2_), 1710 (CO), 1638 (C=C), 1452, 1383, 1303, 1269, 1158, 1100, 1055, 949, 816.

#### Ethyl 2-(12-hydroxy-2-oxadodecyl)acrylate (2d)

bp 129–140 °C/0.039 mbar. IR (film, cm^−1^): 3425 (OH), 2926/2855 (CH_3_/CH_2_), 1714 (CO), 1638 (C=C), 1459/1375/1303 (CH_3_/CH_2_), 1270/1172/1102/1031/949. ^1^H NMR (250 MHz, CDCl_3_, ppm): δ = 1.08–1.24 (m, 15H, C*H_2_*,C*H_3_*), 1.24–1.49 (m, 4H, CH_2_), 3.17 (broad s, 1H, OH), 3.27 (t, 2H, OCH_2_) 3.37 (t, 2H, OCH_2_), 3.96 (s, 2H, CH_2_(1)), 4.01 (q, 2H, OC*H_2_*CH_3_) 5.65 (s, 1H, CH=C), 6.07 (s, 1H, CH=C). ^13^C NMR (63 MHz, CDCl_3_, ppm): δ = 13.64 (CH_3_), 25.36, 25.68, 28.98, 29.07, 29.11, 29.17 and 32.24 (CH_2_ (8-15)), 60.10 (CH_2_OH), 68.31 (O*C*H_2_CH_2_), 70.52 (=C*C*H_2_O), 124.69 (*C*H_2_=C), 137.12 (*C*=CH_2_), 165.37 (CO).

#### Ethyl 2-(14-hydroxy-2-oxatetradecyl)acrylate (2f)

Purification was performed by recrystallization from CH_2_Cl_2_ followed by column chromatography on silica gel (115 g) with CH_2_Cl_2_ as eluent. IR (film, cm^−1^): 3412 (OH), 2924/2855 (CH_3_/CH_2_), 1715 (CO), 1638 (C=C), 1460/1375/1303 (CH_3_/CH_2_), 1270/1173/1103/1054/1029/951.

#### Ethyl 2-(7-hydroxy-2,5-dioxaheptyl)acrylate (2g)

bp 94–102 °C/0.026 mbar. IR (film, cm^−1^): 3426 (OH), 2871 (CH_3_/CH_2_), 1712 (CO), 1639 (C=C), 1456, 1374, 1303, 1270, 1174, 1099, 1028, 953, 887, 816.

#### Ethyl 2-(10-hydroxy-2,5,8-trioxadecyl)acrylate (2h)

bp 138–141 °C/0.041 mbar. IR (film, cm^−1^): 3433 (OH), 2964/2929/2874 (CH_3_/CH_2_), 1711 (CO), 1638 (C=C), 1453/1385/1302 (CH_3_/CH_2_), 1269/1151/1099/1056/984/882. ^13^C NMR (63 MHz, CDCl_3_, ppm): δ = 13.0 (CH_3_), 60.3 (CH_2_O), 68.0–71.5 (CH_2_), 124.3 (*C*H_2_=C), 136.1 (*C*=CH_2_), 164.5 (CO).

#### Ethyl 2-(13-hydroxy-2,5,8,11-tetraoxatridecyl)acrylate (2i)

bp 147–153 °C/0.037 mbar. IR (film, cm^−1^): 3370 (OH), 2870 (CH_3_/CH_2_), 1713 (CO), 1639 (C=C), 1458/1375 (CH_3_/CH_2_), 1266 (CH_2_O), 1198/1028/947/816.

#### Ethyl 2-(5-phosphonooxy-2-oxapentyl)acrylate (3b)

The compound was obtained as a clear, colorless oil. IR (film, cm^−1^): 2926/2855 (CH_3_/CH_2_), 1715 (CO), 1639 (C=C), 1461/1375 (CH_3_/CH_2_), 1265/ 1169/1101/1023/951.

#### Ethyl 2-(8-phosphonooxy-2-oxaoctyl)acrylate (3c)

The compound was obtained as a clear, slightly yellow oil. IR (film, cm^−1^): 2934/2872 (CH_3_/CH_2_), 1714 (CO), 1640 (C=C), 1456/1376 (CH_3_/CH_2_), 1269/1170/1098/1027/950. ^1^H NMR (250 MHz, CD_3_OD, ppm): δ = 1.20–1.36 (t, 3H, CH_3_), 1.43–1.44 (m, 4H, CH_2_), 3.30 (s, 2H, CH_2_), 3.51 (t, 2H, CH_2_), 3.97–3.98 (m, 2H, CH_2_), 4.99 (m, 6H, CH_2_OPO_3_H_2_, OCH_2_CH_3_), 5.86 (s, 1H, CH=C), 6.32 (s, 1H, CH=C). ^13^C NMR (63 MHz, CD_3_OD, ppm): δ = 13.6 (CH_3_), 26.5/26.9/30.6 (CH_2_), 31.4 (*C*H_2_CH_2_OP), 31.5 (OCH_2_*C*H_2_), 60.8 (CH_2_O), 68.0 (CH_2_OP), 70.1 (O*C*H_2_CH_2_), 71.8 (=C*C*H_2_O), 126.4 (*C*H_2_=C), 139.8 (*C*=CH_2_), 169.5 (CO). ^31^P NMR (CD_3_OD; ppm): −0.36/0.14 (d, PO_3_H_2_).

#### Ethyl 2-(12-phosphonooxy-2-oxadodecyl)acrylate (3d)

The compound was obtained as a yellowish solid. IR (film, cm^−1^): 2926/2855 (CH_3_/CH_2_), 1715 (CO), 1639 (C=C), 1461/1375 (CH_3_/CH_2_), 1265/1169/1101/1023/951. ^1^H NMR (250 MHz, CDCl_3_, ppm): δ = 1.15–1.36 (m, 15H, CH_3_), 1.45–1.77 (m, 4H, CH_2_), 3.41 (t, 2H, CH_2_(3)), 4.03–4.24 (m, 4H, C*H_2_*OPO_3_H_2_, OC*H_2_*CH_3_), 4.11 (s, 2H, CH_2_), 5.79 (s, 1H, CH=C), 6.22 (s, 1H, CH=C). ^13^C NMR (63 MHz, CDCl_3_, ppm): δ = 13.98 (CH_3_), 25.08, 25.93, 28.92, 29.19, 29.25, 29.30 and 29.45 (CH_2_ (8-15)), 60.42 (*C*H_2_CH_3_, *C*H_2_OP), 68.63 (O*C*H_2_CH_2_), 70.86 (=C*C*H_2_O), 125.08 (*C*H_2_=C), 137.38 (*C*=CH_2_), 165.69 (CO).

#### 2-(12-phosphonooxy-2-oxadodecyl)acrylic acid (3e)

To an ice cold solution (0–5 °C) of 3.470 g (10.14 mmol) of **3d** dissolved in 20 mL water, an aqueous solution of 1.217 g (30.41 mmol) sodium hydroxide dissolved in 12.165 mL of water was added slowly. The solution was then stirred for 24 h at ambient temperature. The basic solution was acidified with 25 mL of a 1N HCl, saturated with NaCl and extracted three times with 50 mL THF. The extracts were dried over Na_2_SO_4_ and the solvent was removed. Yield: 2.01 g (62.8%), of a yellowish solid, mp 63.2 °C

IR (film, cm^−1^): 3500–3100 (COOH, broad), 2924/2854 (CH_3_/CH_2_), 1697 (CO), 1633 (C=C), 1458/1373 (CH_3_/CH_2_), 1173/1010/827. ^1^H NMR (250 MHz, CDCl_3_, ppm): δ = 1.95–2.18 (m, 16H, CH_2_), 3.90 (t, 2H, C*H_2_*OPO_3_H_2_), 4.37–4.43 (m, 2H, OC*H_2_*CH_2_), 4.6 (s, 2H, =CCH_2_O), 5,75 (s, 1H, CH=C), 6.30 (s, 1H, CH=C). ^13^C NMR (63 MHz, CDCl_3_, ppm: δ = 25.08–31.6 (CH_2_), 67.3 (CH_2_OP), 69.5 (O*C*H_2_CH2), 71.2 (=C*C*H_2_O), 125.8 (*C*H_2_=C), 137.6 (CH_2_=*C*), 168.7 (CO).

#### Ethyl 2-(14-phosphonooxy-2-oxatetradecan)acrylate (3f)

The compound was obtained as a white wax-like solid. IR (film, cm^−1^): 2918/2854 (CH_3_/CH_2_), 1712 (CO), 1637 (C=C), 1470/1379 (CH_3_/CH_2_), 1304/1238/1165/1116/1026/853. ^1^H NMR (250 MHz, CD_3_OD, ppm): δ = 1.28–1.41 (m, 17H, CH_3_), 1.58–1.69 (m, 4H, CH_2_), 3.48-3.51 (t, 2H, CH_2_), 3.94–3.98 (q, 2H, OC*H_2_*CH_3_), 4.15 (s, 2H, CH_2_), 5.20 (s, 2H, CH_2_), 5.85 (s, 1H, CH=C), 6.26 (s, 1H, CH=C). ^13^C NMR (63 MHz, CD_3_OD, ppm): δ = 15.50 (CH_3_), 26.69, 27.31, 30.37, 30.61, 30.75, 31.49 and 31.56, (CH_2_), 62.52 (*C*H_2_CH_3_), 67.81, 67.69 (CH_2_OP), 70.21 (O*C*H_2_CH_2_), 71.99 (=C*C*H_2_O), 126.28 (*C*H_2_=C), 139.51 (*C*=CH_2_), 169.51 (CO). ^31^P NMR (CD_3_OD; ppm): −0.57/0.13 (d, PO_3_H_2_).

#### Ethyl 2-(7-phosphonooxy-2,5-dioxaheptyl)acrylate (3g)

The compound was obtained as a clear, colorless oil. IR (film, cm^−1^): 2926/2878 (CH_3_/CH_2_), 1712 (CO), 1640 (C=C), 1453/1379 (CH_3_/CH_2_), 1240/1169/1105/818. ^1^H NMR (250 MHz, CD_3_OD, ppm): δ = 1.30–1.38 (m, 3H, CH_2_C*H_3_*), 1.70–1.85 (m, 2H, CH_2_), 3.31–3.46 (t, 2H, CH_2_), 3.61–3.73 (m, 4H, OC*H_2_*CH_3_), 4.07–4.25 (m, 2H, CH_2_), 4.88 (s, 2H, CH_2_), 5.92 (s, 1H, CH=C), 6.27 (s, 1H, CH=C). ^13^C NMR (63 MHz, CD_3_OD, ppm): δ = 14.47 (CH_3_), 61.88 (*C*H_2_CH_3_), 66.89/66.73 (CH_2_OP), 70.4 (CH_2_*C*H_2_O), 70.48 (O*C*H_2_CH_2_), 71.42 (=C*C*H_2_O), 71.49 (O*C*H_2_CH_2_), 126.65 (*C*H_2_=C), 139.01 (*C*=CH_2_), 164.72 (CO). ^31^P NMR (CD_3_OD; ppm): −0.01/0.79 (d, PO_3_H_2_).

#### Ethyl 2-(10-phosphonooxy-2,5,8-trioxadecyl)acrylate (3h)

The compound was obtained as a clear, colorless oil. IR (film, cm^−1^): 2874 (CH_3_/CH_2_), 1711 (CO), 1639 (C=C), 1459/1372 (CH_3_/CH_2_), 1246/982/820. ^1^H NMR (250 MHz, CD_3_OD, ppm): δ = 1.28–1.32 (m, 3, CH_3_), 3.67/3.72 (m, 10H, CH_2_), 4.09–4.10 (q, 2H, OC*H_2_*CH_3_), 4.21–4.23 (t, 2H, CH_2_), 5.34 (s, 2H, CH_2_), 5.91 (s, 1H, CH=C), 6.30 (s, 1H, CH=C). ^13^C NMR (63 MHz, CD_3_OD, ppm): δ = 13.57 (CH_3_), 61.95 (*C*H_2_CH_3_), 66.86/66.91 (CH_2_OP), 70.4 (CH_2_*C*H_2_O), 70.5 (O*C*H_2_CH_2_), 71.19 (=C*C*H_2_O), 71.60 (O*C*H_2_CH_2_), 126.74 (*C*H_2_=C), 139.21 (*C*=CH_2_), 165.6 (CO). ^31^P NMR (CD_3_OD; ppm): 0.81/0.01/−0.81 (m, PO_3_H_2_).

#### Ethyl 2-(13-phosphonooxy-2,5,8,11-tetraoxatridecyl)acrylate (3i)

The compound was obtained as a clear, colorless oil. IR (film, cm^−1^): 2879 (CH_3_/CH_2_), 1714 (CO), 1639 (C=C), 1465/1398 (CH_3_/CH_2_), 1258/1150/1026/796. ^1^H NMR (250 MHz, CD_3_OD, ppm): δ = 1.21–1.28 (m, 3, CH_3_), 3.59/3.63 (m, 14H, CH_2_), 4.06–4.14 (q, 2H, OC*H_2_*CH_3_), 4.21–4.23 (t, 2H, CH_2_), 5.34 (s, 2H, CH_2_), 5.92/5.94 (s, 1H, CH=C), 6.24/6.28 (s, 1H, CH=C), 8.71 (s, 2H, PO_3_H_2_). ^13^C NMR (63 MHz, CDCl_3_, ppm): δ = 13.7 (CH_3_), 60.5 (*C*H_2_CH_3_), 65.5 (CH_2_OP), 68.8 (CH_2_CH_2_O), 69.3 (O*C*H_2_CH_2_), 69.7 (=C*C*H_2_O), 127.9 (*C*H_2_=C), 136.4 (*C*=CH_2_), 168.5 (CO). ^31^P NMR (CD_3_OD; ppm): −0.11/0.07 (d, PO_3_H_2_).

Adhesive composition **4a**: 1.5261 g *N*,*N*´-bisacrylamido-1,3-propane, 2.2150 g 3,(4),8,(9)-bis(acrylamidomethyl)tricyclo[5.2.1.0^2,6^]decane, 0.5551 g of **3a**, 0.2776 g 2-acrylamido-2-methylpropanesulfonic acid, 0.0337 g camphorquinone, 0.0849 g bis(2,4,6-trimethylbenzoyl)phenyl phosphine oxide and 0.0392 g 4-(*N*,*N*-dimethylamino)benzoic acid ethyl ester were dissolved in a solvent mixture of 3.5201 g ethanol and 1.7484 g water.

Adhesive compositions **4c–i** were prepared from **3c–i** in the same manner as described above.

The following procedure was applied prior to adhesion measurements:

- First the teeth were abraded with 200 and 500 grit abrasive paper.
- Then the teeth were stored at 37 °C in water.
- The teeth were treated with adhesive formulation for 20 s and the solvents evaporated by an air stream for 10 s.
- Light curing of adhesive layer for 20 s.
- A polymerized Spectrum TPH body applied on the adhesive was cured on the teeth 3 times for 20 s.
- Finally, the prepared teeth were stored in water at 37 °C for 2 h before the measurement.

IR spectra were measured with a FT-IR spectrometer (Nicolet 6700 FT-IR spectrometer, Thermo Scientific). The ^1^H NMR, ^13^C NMR and ^31^P NMR spectra were obtained with a Bruker AC 250 MHz spectrometer. Viscosities were measured with a Bohlin-Rheometer CS-50 at 23 °C.

The melting points were taken with a DSC 7 (Perkin-Elmer). Polymerization enthalpies were measured in the isothermal mode at 37 °C with a photo-calorimeter DSC 7/DPA 7 (Perkin-Elmer). The light intensity in the visible portion of the spectrum was 108 mW**^.^**cm^−2^. Each DSC experiment included a short dark period (typically 6 s) and a subsequent illumination period. After the first run, an additional run was made using the polymerized material under the same experimental conditions. The subtraction of these runs from one another removed the effect of different baselines for the dark and the illumination periods. In the monomers were dissolved 0.3 mol % camphorquinone and 0.35 mol % 4-(*N*,*N*-dimethylamino)benzoic acid ethyl ester.
